# Do Two Machine-Learning Based Prognostic Signatures for Breast Cancer Capture the Same Biological Processes?

**DOI:** 10.1371/journal.pone.0017795

**Published:** 2011-03-14

**Authors:** Yotam Drier, Eytan Domany

**Affiliations:** Department of Physics of Complex Systems, Weizmann Institute of Science, Rehovot, Israel; Vanderbilt University Medical Center, United States of America

## Abstract

The fact that there is very little if any overlap between the genes of different prognostic signatures for early-discovery breast cancer is well documented. The reasons for this apparent discrepancy have been explained by the limits of simple machine-learning identification and ranking techniques, and the biological relevance and meaning of the prognostic gene lists was questioned. Subsequently, proponents of the prognostic gene lists claimed that different lists do capture similar underlying biological processes and pathways. The present study places under scrutiny the validity of this claim, for two important gene lists that are at the focus of current large-scale validation efforts. We performed careful enrichment analysis, controlling the effects of multiple testing in a manner which takes into account the nested dependent structure of gene ontologies. In contradiction to several previous publications, we find that the only biological process or pathway for which statistically significant concordance can be claimed is cell proliferation, a process whose relevance and prognostic value was well known long before gene expression profiling. We found that the claims reported by others, of wider concordance between the biological processes captured by the two prognostic signatures studied, were found either to be lacking statistical rigor or were in fact based on addressing some other question.

## Introduction

Technological advances made during the last decade have allowed measurement of enormous amounts of molecular data from a tumor tissue resected from a particular subject. The main challenge of modern cancer research is bridging the gap between these data and clinically significant questions that need urgent answers, such as prognosis and prediction of response to therapy.

The first issue, of prognosis, is highly relevant, since it is used to decide whether to subject a patient to chemotherapy. This decision is extremely important for the individual as well as for society for three main reasons. First, nearly all available chemotherapy is detrimental to the patient, since it adversely affects healthy tissue as well as the malignant one, at which it is aimed. Second, some of the side effects, even if they do not have a direct impact on the patient's physical well-being, may cause considerable psychological damage and hardship. Finally – chemotherapy is extremely expensive.

It is well known that for many cancers prognosis and the need for therapy may vary widely; while in some cases surgery and adjuvant radiotherapy suffice to eradicate the disease, other tumors are very aggressive, will recur, metastase and kill the patient. While aggressive tumors call for chemotherapy, overtreatment of “good outcome” patients by administering unneeded chemotherapy is, unfortunately, very common. This is the case particularly in breast cancer, where increased awareness has brought, through regular frequent checkups, a considerable increase in the number of early discovery cases, of small tumors of low stage and grade.

It is believed that the currently accepted clinical-pathological criteria for administering chemotherapy gives rise to overtreatment of a very large fraction of early discovery breast cancer patients. Therefore, there is an acute need for reliable biomarkers that can, on the basis of measurements done on the primary tumor tissue, differentiate poor from good outcome.

A large number of methods have been introduced to generate biomarkers from available molecular information (in particular from gene expression microarray data – see [1], [2], [3], [4] for reviews). Two prognostic platforms based on expression signatures are commercially available: OncotypeDx, based on a 21-gene signature measured on paraffin-embedded samples by polymerase chain reaction (PCR) [5], and Mammaprint, the 70-gene “Amsterdam signature” measured by a microarray [6], [7], [8], [9].

Considerable criticism has been raised about the following aspects of several proposed signatures: lack of robustness, various statistical and machine-learning related problems, low success rates for the cases that are hard to prognosticate by existing methods, and lack of biological meaning of gene lists, that were obtained without biological guidance.

The first criticism, concerning the statistical validity and robustness of the reported gene lists, focuses on the fact that in many cases the reported signatures were derived and tested in only one particular way, which was arbitrarily selected out of many equally legitimate ones. For example, one can split the samples into a training set and a test set in a combinatorially large number of ways. Hence the entire analysis, including training, gene selection and testing, can be repeated many times, using the same data, but splitting differently the samples into training and test sets. Each such split can be viewed as a particular instance of the analysis, and by performing many such repeats, one can generate distributions of various quantities of interest. In particular, one can calculate for each split the success rate, defined as the fraction of successful predictions of outcome on the test set, and estimate the distribution of the success rates by repeating the analysis many times. Once this distribution is known, one can estimate the probability to find a success rate as good as, or better, than the one reported in the actual published study (for which the analysis was repeated). When this was done [10], [11], the results of many studies have been demonstrated to be “overoptimistic” [11]; the success rate that was actually reported had a much lower than acceptable probability of being observed. The overoptimistic reported success rates of many studies were explained by falling into various statistical pitfalls [2], [12]. These include severe overtraining [2], due mainly to “information leak” which has been explicitly identified in a number of cases [2], [13]. The term information leak refers to allowing usage of any information about the test set during the training phase. Another issue concerns the *prognostic lists* of genes (which are the ones that are actually placed on a prognostic device [7]). The genes that appear in the prognostic list of a particular study were selected by ranking all the tested genes (for example, on the basis of the correlation of their expression values (measured over the training samples) with outcome. These lists were shown to lack robustness [10] for the sample sizes used [14], [15]; i.e. the prognostic gene lists changed almost completely when the procedure was repeated. It has been shown [14], [15] that if a training set of ∼100 early breast cancer samples is used to rank ∼10,000 genes (by their correlation with outcome), and the ∼100 top genes are selected as the prognostic set, repeating the procedure (with a different set of training samples) will produce a new gene list, whose overlap with the first one is typically 2–3%. Since the different gene lists obtained even from the same particular study are very unstable against repeating the analysis, one clearly expects even less overlap between lists produced by different studies (in which different patients, different microarray facilities and even different platforms were used). In response to this criticism it was stated that if two divergent lists provide concordant prognostication and acceptable success rates, one should not care about their lack of robustness [16]. This response was countered, however, by criticism raised against the criteria that were used to assess the success rates of several expression-based classifiers [17], and various publications questioned whether they actually performed better than either a single-gene based classifier [18] or one that uses classical clinical and pathological indicators [19], [20]. The issue of concordance [21] or lack thereof [17], [22] between different prognostic signatures was also debated.

The points of criticism described above address either technical issues that concern the standard machine-learning approaches taken by most derivations of prognostic signatures, or the clinical utility of the resulting classifiers. In the present study we focus on a third type of criticism, directed at the lack of biological meaning of various prognostic signatures. Some signatures [5], [23], [24], [25], [26], [27] did use biological and clinical knowledge to assemble their predictive genes. We did not consider the Oncotype DX recurrence signature [28], which was constructed by carefully picking genes from relevant pathways (and therefore indeed, capture many pathways); the P53 signature [24], BMI1 signature [27] and wound response signature [23], [29], each of which was constructed to capture a specific pathway, as their names suggest (and therefore indeed mainly capture the desired pathway); or the genomic grade signature [30] that was constructed specifically to capture histological grade (and was found to include mostly proliferation-related genes [31]). Our focus is on prognostic gene lists which were derived using the “top-down” approach as defined in [32], that is, either using no biological guidance at all for feature selection and training – e.g. the Amsterdam signature [7], or using very minimal biological input, such as for the 76-gene Rotterdam signature [33], which treated ER+ and ER− breast cancers separately.

According to the critics, these prognostic gene lists lack clear biological interpretation and probably contain no biologically relevant discovery. In response to this criticism it was claimed by some [34] that the biological processes that were represented by the activities of the genes on such divergent lists did, in fact, exhibit considerable similarity. If correct, this claim gives one more reason why one should not worry about the fact that the gene lists of different studies had no overlap; furthermore, this would also answer the criticism regarding biological meaning.

The claim that divergent gene lists from different studies do reflect the activities of similar cancer-related pathways and biological processes seems to be advocated and accepted by many [1], [9], [15], [21], [34], [35], [36]. Only a few studies [37], [38], [39], [40] have, however, actually tried to substantiate these claims in a quantitative manner. The aim of our study is to test the validity of these claims in a way which we believe is conceptually and statistically sound.

In what follows, we first present the guiding principles that must be adhered to in order to test properly these claims, and then we review critically the studies mentioned above. Next we present our results obtained when the analysis is carried out for two important signatures [7], [33] according to our guiding principles. We conclude that the only biological processes and pathways that are significantly represented by both these signatures are cell proliferation and its variants.

### The guiding principles of the present study

Our aim here was to test critically the claims that two different machine-learning based prognostic gene lists capture similar biological processes. To this end we examined the two most established outcome prediction signatures, the 70 gene list of van't Veer et al. [7] and the lists defined by Wang et al. [33], both the 60 gene ER+ signature and the complete 76-gene list. We have chosen these two signatures as they were learned independently and without forcing specific biological pathway-based knowledge.

We adopted the following guiding principles in designing our test:

1. Use only the genes that actually appear in the prognostic lists.
2. Identify over-represented biological processes by means of enrichment analysis.
3. Address the problem of false discoveries generated by multiple comparisons that are made, but take into account all the dependencies and nested structures present in the ontologies used.
4. Use more than one gene ontology, to minimize dependence on incomplete or deficient class assignments.

The rationale for the first principle is the following. As stated above, our aim is to test, in a statistically correct way, the claim that was voiced by proponents of the proposed prognostic lists, that different lists do capture the same biological processes. To test this claim, one is not supposed to use larger gene lists, which could have been derived from the same experiment by some other means. We are neither claiming that gene expression cannot possibly capture important and biologically relevant prognostic information, nor are we attempting to demonstrate how one could, in principle, capture such information.

In fact it is likely that the full data gathered in these studies do reflect similar deregulation of a few common relevant pathways, but it remains to be proven that this similarity is captured in the actual proposed gene lists. In that regard it is worth mentioning that when standard machine-learning methods are used to select features (genes) for a classifier, the number of selected features cannot exceed significantly the number of samples that happened to be available for training [41] (at the time when the study was first performed and the gene lists were selected). Otherwise the classifier is trained to recognize the noise in the particular training set used, and will fail on any test set (since while the true “signal” is the same in the training and test sets, the noise is completely independent). This limitation might restrict the selected number of genes and produce lists of selected genes that are too short to capture the necessary biological processes. Two possible ways to overcome this are producing much longer gene lists (for which much more training samples must become available), or use biologically relevant knowledge based considerations to select the predictive genes.

The second principle states that a generally accepted method [42] be used to assess enrichment of a pathway or biological process by the prognostic list.

The third principle – the necessity for taking into consideration the false discoveries [43], [44] that arise when multiple comparisons are made - cannot be overemphasized [41]. A problem arises when one performs enrichment analysis of GO (gene ontology) terms [45], such as Biological Processes (GOBP). When the number of GO terms is taken as the number of independent tests, it is likely that not a single term will pass any of the available procedures [46] that control the FDR. The reason is that because of the nested and overlapping structure of the ontology, the many terms tested are not independent and hence the standard methods that control the FDR are much too stringent [47], [48] (to understand this point, imagine that in fact we have one single term which for some reason is repeated 1000 times – while only a single test was performed, naively we may think that 1000 hypotheses were tested). The trivial resolution of this problem, of ignoring multiple testing altogether and make no attempt to control the FDR, goes to the opposite extreme and is way too permissive, generating a very large number of false positive apparently enriched GO terms.

We present and compare three ways to deal with the problem of multiple comparisons. The first is to apply the standard Benjamini-Hochberg procedure [43] to control the FDR, ignoring the nested structure of the ontologies. We show that this procedure, which is probably too stringent, finds almost no commonly enriched biological processes or pathways. The second and third are two different ways, explained in detail in the Methods section, designed to deal with multiple comparisons while taking the dependencies and nested structure of the ontologies into account.

The fourth principle stems from the known fact that ontologies are far from being perfect, and probably contain some incorrectly assigned genes; testing a claimed enrichment for more than one ontology or database is prudent.

The manner in which each of these points is implemented is explained in detail in the Methods section below.

### Brief review of previous work

The abstract of Yu et al. [40] states that “We show that divergent gene sets classifying patients for the same clinical endpoint represent similar biological processes …”. They addressed this issue indirectly by using expression data of 344 early discovery breast cancer patients; the same analysis was done separately for the ER+ and ER− cases. 80 samples were selected at random as training set; Cox regression analysis was performed to identify the 100 genes whose expression was most correlated with distant metastasis-free survival time. These “top 100” genes were analyzed for enrichment of 304 GOBP (selected, using some arbitrary thresholds, from the total list of GOBP). The enrichment analysis was done as follows: hypergeometric p-values were calculated (Fisher's exact test) for over-representation of the genes that belong to a GOBP among the 100 “top genes”, and if the number of genes exceeded one and the p-value was less than 0.05, the GOBP was declared enriched. No correction for multiple comparisons (of either genes or GOPB) was used, and no special treatment to the GOBP dependence (due genes that appear in several GOBPs) was offered. This analysis was repeated 500 times, yielding 500 lists of enriched GOBP. The 20 GOBP that had the highest number of appearances were assembled, for ER+ and ER−, yielding 36 “core pathways” (4 appeared on both lists). Finally, several published prognostic gene lists were analyzed for enrichment among the 304 GOBP and among the 36 core pathways, and using the hypergeometric distribution, significant overrepresentation of the core pathways was reported.

This analysis is too permissive mainly because no FDR correction for multiple comparisons was used at all. Moreover, several arbitrary and unjustified thresholds were used for selection of GOBP to be tested and for identification of enriched GOBP; the sets of enriched GOBPs obtained for each pair of prognostic gene lists were not compared directly, but each was compared to the list of core pathways defined above; only one database of biological pathways and processes was used for the study.

Shen et al. [38] have followed similar guidelines to those we suggest. They actually don't find a statistically significant number of pathways common to the Wang and van't Veer lists (this fact is not emphasized, but see Figure 1 of their paper). Moreover, the statistical significance of the overlaps they report is due to an unusual definition of the p-value. Namely, if they find that the tested prognostic list contains k genes from a pathway, they estimate the p-value as the probability that a random gene list will contain more than k genes from the pathway- p(x>k), instead of using the standard definition, i.e. the probability to find k or more than k such genes- p(x≥k). These two probabilities are nearly the same for most situations, but can be quite different when the list is very short (small k), as is the case here, where often k = 1. Table 4 in their paper shows what appears to be significant overlap between several signatures, but in fact there is only one single gene of the 70 gene list that belongs to each of the ‘enriched’ pathways. Given that 50 genes from the 70 are annotated, chosen out of 11342 genes on the chip (the “population”), and that, for example, the RECK pathway (one of the five presented as significantly over-represented and shared in Table 4) has 8 genes from the population, a naïve hypergeometric test will conclude a p-value of 0.035, while Shen's measure will indicate a much higher significance, of 5.24*10-4. Checking the hypothesis for all probes (not just annotated ones) will increase the p-value further. The naïve hypergeometric high p-values will not pass a reasonable FDR on the 552 hypotheses checked. The other 4 pathways also have only one gene among the 50, and since these pathways contain more genes than RECK, their p-values will only be bigger. Even if one chooses to ignore the 70 gene list, and look for pathways common only to the three other signatures checked in the paper, only the breast cancer estrogen signaling pathway is found to be over represented in all. Repeating this analysis using the standard definition of the p-value, we found that for the 70 gene list no pathway passes at any reasonable FDR, and even if we ignored the 70 gene list, still only the breast cancer estrogen signaling pathway was over represented in all the other three signatures tested.

Reyal et al. [37] have not approached the question of pathway convergence of the signatures directly, but instead aimed at offering a new, pathway based predictor. In order to do so they have used a large number of tumor expression profiles measured by the Affymetrix 133A platform, started from seven published signatures and used them to create enlarged signatures. These contained all the genes correlated to the original signature, revealing large gene clusters that differentiated good from bad outcome. A careful pathway analysis discovered common pathways which were then used to build new, more promising predictors. In our context, however, one must be careful not to deduce from this study that there is biological agreement between the actual seven signatures they studied, as their analysis was done on highly enlarged gene lists.

Sole et al. [39] have tested different signatures of different cancers, including breast cancer signatures [5], [29], [49], [50], [51], [52], by two main approaches. The first was to check for overrepresentation of transcription factor targets as predicted by motif analysis and chip experiments. The second was to check on a few datasets for correlations between the signature genes and the various pathway genes. The first approach identified targets of E2F and ER, as well as cell cycle genes, to be common to many of the signatures. Note that E2F is a major proliferation regulator and many of its targets correlate with proliferation rate. They have raised also the possibility that AHR, MYB and MYC targets are overrepresented in a few of the signatures. The second approach identified mitosis and possibly immune response as related to some of the breast cancer signatures on the examined data. Note that the second approach may reflect the prognostic potential of the found pathways, but not the biological convergence of the signatures.

## Methods

### Compared prognostic signatures

van't Veer's signature was developed based on ∼5000 probes (we reproduced a list of 5159 probes) from the Rosetta Hu25K microarray, Wang's signature was developed based on 17816 probes from the Affymetrix U133A microarray. These probes were selected by filtering out probes with low signal, and hence were the actual candidates for the signature, and therefore we chose these lists as the background references.

The Hu25K probes were matched to known genes by their sequences using BLAST [53], and mapped into official gene symbols. We used Affymetrix's mapping of the U133A probe sets to gene symbols. For TANGO analysis probes were converted to Entrez GeneID using MatchMiner [54]. Since not all probes capture a recognized gene with an official gene symbol, and some probes capture more than one gene, the actual lengths of the lists are slightly different than the corresponding list of probes. This, however, does not affect the enrichment analysis as probes with no recognized official gene symbol also have no known annotations. The gene lists of the van't Veer and Wang signatures are listed in Table S1.

### Testing for significant pathway enrichment of each list with standard FDR control

The pathway databases used for our analysis are the Gene Ontology Biological Process [45] annotations, (as downloaded from [55]) and MSigSB C2 Canonical Pathways database (version 2.5) [56], which integrates 12 different pathway databases. When referring to a GOBP annotation we refer to all the genes in this annotation together with all the genes of all the descendent annotations. Only annotations that had at least one gene in the relevant background were considered. When considering the size of the annotation, only genes that appear in the relevant background were counted.

For every individual gene list (signature) studied we tested enrichment by genes that belong to a particular biological process or pathway. Enrichment of annotations were computed by Fisher's exact test, using for each signature as background reference the gene population of the original experiment from which the signature was derived (i.e. genes from the corresponding chip that have passed the initial filtering), and correcting for multiple testing by standard control of the FDR [43], without taking the nested dependencies of the GO annotations into account.

### More accurate control of multiple hypotheses, using resampling

One might claim that using the standard methods to control the FDR is too strict (mainly due to the dependencies between the pathways). Alternative approaches were suggested to test for annotation enrichments, which were claimed to be less stringent than the standard control of FDR, while still offering correction for testing multiple hypotheses. TANGO [48] performs functional enrichment tests that fully account for multiple testing, using a simple resampling algorithm. The aim is to assess the significance of the enrichment of a gene set *T* in the different biological processes *A_i_* of an ontology *A*. First, TANGO computes the hypergeometric p-values *p_i_* of *T* against all the processes. To determine (in a way that takes multiple testing into account) which of these is significant (at say 5% level), TANGO calculates the empirical background distribution of the best p-values obtained for each one of a large number of randomly generated gene lists (of the same length as *T*). Finally, the corrected p-value of each process *A_i_* is determined as the probability to do better, using the background distribution. This way all the relations among the biological processes of the ontology are preserved.We have used the EXPANDER [57] implementation of TANGO to test for GO annotation enrichments in all three lists (Wang 60, Wang 76, and van't Veer 70), and determined the threshold on the corrected p-values one needs to use in order to have even a single enriched process shared by van't Veer and one of the Wang lists.

### Correcting pathway overlap for multiple testing by assessing the significance of shared processes

At the opposite end of the spectrum of stringency one is ignoring the problem of multiple hypotheses and simply looks for biological processes that passed some threshold on the enrichment p-value *for both gene lists*, such as performing Fisher's exact test [42] and taking only p-values smaller than 0.05. Clearly, since the set of biological processes that satisfied this criterion was derived neglecting completely multiple testing (e.g. of testing many biological processes for enrichment), this procedure is too permissive. To estimate the significance of the fact that a biological process passed this criterion in a way that corrects for multiple testing, we devised a random model to generate a relevant background distribution, which takes into account the real dependences between the pathways and biological processes. Two random lists, *L60* and *L70*, containing 60 and 70 genes, were generated from the respective lists of probes from the chips used by van't Veer and by Wang. We then preformed Fisher's exact test between the genes that correspond to the selected probes of each of the two lists and every biological process (or pathway), and determined the number *x* of processes with enrichment p-value smaller than some threshold *q* (we used *q* = 0.05, 0.10 and 1.0), for *both L60* and *L70* (as opposed to *TANGO* which estimates enriched pathways for every list). By repeating this process 5000 times and calculating the histogram of *x*, we constructed a background distribution *P(x = k)*, estimating the probability to get by chance *k* processes with hyper-geometric enrichment p-value<*q* for both random gene lists. Hence, the significance of observing *c* biological processes or pathways for which both the Wang and van't Veer gene lists are enriched at this level, is simply estimated by *P(x> = c)*. Note, that just like in the actual process of learning the signatures, probes that do not map to known genes with known annotations could be selected, and therefore the effective length of the gene list is usually smaller.

## Results

### Testing for significant pathway enrichment of each list with standard FDR control

In order to check rigorously the claims of convergent biological pathways and processes for different gene lists, we examined (see Methods) the two most established outcome prediction signatures, the 70 gene list of van't Veer et al. [7] and Wang et al's ER+ signature [33].

We have chosen the richest, well accepted annotation database, the gene ontology biological process (GOBP) database [45] as the major list of pathways, and repeated the analysis with two more lists, for the sake of completeness (see below).

Only a single process, DNA Replication, passed FDR in both signatures, at a very permissive level of 0.31. Raising the bar to FDR = 0.53 gave rise to the microtubule cytoskeleton organization pathway, and with even more permissive FDR only two closely related annotations emerged - ‘microtubule-based process’ and ‘DNA-dependent DNA replication’. This indicates that probably both signatures capture some aspects of cell cycle and proliferation.

It is worth mentioning that the well accepted DAVID annotation tool [58], [59] does not find any enriched pathway in any of the signatures, which passed FDR of 0.9, other than organelle organization and biogenesis in Wang's signature (q = 0.36).

We repeated the analysis for a shorter list of GOBPs along the lines of Yu et al. [40] who tested only those 304 GOBPs that had representative probe sets for at least 10 of their genes on the U133A chip. We found 1373 such GOBPs and repeated our analysis limited to this list (we believe that the discrepancy between 304 and 1374 is due to the fact that Yu et al used a very early version of the GO database). Next, we also examined the MSigDB canonical pathways database [56], collecting metabolic and signaling pathways from 12 online pathway databases. Furthermore, we repeated the analysis for the entire signature of Wang (76 probe sets). All these additional comparisons yielded even less common pathways than the original one. The full results of all the pathways that passed FDR of 0.75 in any one of the three databases are shown in Table S2. Few more details can be found in the Methods section.

### More accurate control of multiple hypotheses, using resampling

TANGO [48], a resampling based method for pathway enrichment analysis (see Methods), did not find any pathways with p-value smaller than 0.48, see Table S2 for annotations with less significant p-values (DNA Replication was found in all signatures, but with a p-value higher than 0.8).

#### Correcting pathway overlap for multiple testing by assessing the significance of shared processes

Using the “p-value smaller than 0.05” criteria with p-values obtained by Fisher's exact test of both signatures (see Methods), gave rise to 18 common pathways, most of which were related to cell cycle. Raising the allowed p-value threshold to 0.1 discovers 10 more pathways of different contexts, as also shown in Table 1.

**Table 1:** The list of pathways whose hypergeometric p-value is less than 0.05 and 0.1, without correcting for multiple hypothesis testing.

Common pathways for p<0.05	Additional common pathways for p<0.1
DNA metabolic process	axon regeneration
DNA packaging	cell cycle
DNA replication	cellular component organization
DNA replication initiation	chromatin assembly or disassembly
DNA strand elongation	intracellular signaling cascade
DNA strand elongation during DNA replication	mitotic cell cycle
DNA-dependent DNA replication	negative regulation of translation
cell division	nucleus localization
chromosome condensation	response to hypoxia
chromosome organization	second-messenger-mediated signaling
cytokinesis during cell cycle	
cytoskeleton organization	
microtubule cytoskeleton organization	
microtubule-based process	
mitotic chromosome condensation	
nucleosome assembly	
organelle organization	
phosphoinositide-mediated signaling	

Since this overlap was derived neglecting multiple testing completely, it is too permissive. We estimated the significance of this overlap using a random model (see Methods) to generate a relevant background distribution that takes into account also the real dependences between the pathways and biological processes. This analysis finds that the number of common Biological Processes (derived without any FDR control) that we found for the real lists was significantly higher than the number for random signatures- we calculated a p-value of 0.015 to get the observed overlap for threshold of p<0.05 (and 0.068 for p<0.1), showing that indeed both signatures capture some common essence. As before, the process was repeated for the reduced GOBP list and MSigDB, as well as for Wang's complete 76 genes signature. The results of the analysis were similar, as shown in Table S3.

### What common pathways are really present, other than proliferation?

The fact that both signatures capture cell cycle and proliferation is evident. It is well known that there are many genes whose mRNA level correlates with proliferation, usually referred to as the “proliferation cluster”, since they are all cluster together [60], [61], [62]. Indeed both signatures contain genes from the proliferation cluster, which enables them to approximately capture the rate of proliferation. To test whether there are any additional common pathways, we omitted from both lists the genes that were highly correlated with cell proliferation. Those genes were identified by calculating the Pearson correlation of their expression with the expression of a gene known to be correlated with the rate of proliferation in the EMC-344 cohort [33], [63]. To be on the safe side, 3 attempts were made, each using a different proliferation gene (either MKI67, TOP2A or CDK1, all of which appear in three major papers discussing the members of the proliferation cluster [60], [61], [62]). For each attempt, all the genes with Spearman correlation of at least 50% were omitted from the list on which they appeared. The genes that were omitted are listed in Table S4; as can be seen, in all cases the common gene cyclin E2 was omitted. The enrichment analyses described above were now repeated for the filtered signatures.

The results of the enrichment analysis have changed dramatically. No common pathways have passed any FDR (as a matter of fact, no pathway was found in the reduced van't Veer signature that passed any FDR<1). Ignoring FDR corrections, only 1–2 common pathways were found with p<0.05 (nucleus localization and in the MKI67 case, also response to hypoxia), and 5 common pathways for p<0.1. These overlaps were found to be not statistically significant when comparing to the generated background distribution, as described above (p-values of 0.6–0.8). As before, the process was repeated for the reduced GOBP list and MSigDB, as well as for Wang's complete 76 genes signature, yielding similar results. For more details see Table S5 and Table S6.

One might claim that pathway enrichment is not an accurate enough tool to answer the question whether two signatures capture the same biological features. This might be true, but in this case some other proof is necessary, and none has been presented yet. For example, possibly the presence of even one single gene from a pathway could suffice to capture a biological feature, at least to some extent. Proliferation is a good example of such a possibility, since apparently any gene picked from the proliferation cluster will capture the proliferation rate. If this is indeed the case, our test would find an insignificant enrichment, while in fact the pathway *is* represented to some extent. It seems hard to believe, however, that the expression level of one gene can capture the level of activity or deregulation of more complex pathways.

## Discussion

We presented a comprehensive analysis aimed at answering the question whether the two outcome prediction signatures for early-discovery breast cancer, of van't Veer et al and Wang et al, capture the same biological processes. We focused on these two signatures since they were derived using machine learning approaches, with minimal biological knowledge incorporated in the choice of the predictive genes. While such an overlap between the biological processes has been claimed or implied, very few studies have actually tested this claim. We performed our tests in a way that on the one hand did not ignore the problems of multiple testing, but on the other hand took into account the dependent and nested nature of the gene ontologies used. We found that the concordance of enriched pathways between the two tested signatures is restricted to capturing the cells' proliferation rate. When proliferation-related genes are deleted from the two lists, the number of pathways over represented in both signatures does not exceed the number of such pathways expected for two random gene lists.

Taken together, all the results obtained indicate that while there is some common biology captured by the two signatures, it is very limited: all the processes captured by both signatures are related to cell proliferation.

To conclude on a constructive note, we do believe that an expression-based prognostic method that is knowledge-based, i.e. one that incorporates also well-established biological and clinical information on relevant pathways, will be able to improve current prediction capabilities.

## Supporting Information

Table S1 Gene symbols of the genes in van't Veer and Wang signatures used in the analysis. The Wang signatures were converted from the published probe sets by Affymetrix official tables. The van't Veer signature was converted from the published probes using BLAST. The common gene cyclin E2 is highlighted.
(XLS)

Table S2 Enrichment analysis of each signature separately. FDR controlled hypergeometric enrichment of van't Veer signature, Wang 60 gene ER+ signature and Wang 76 gene signature, for GOBP annotations (both complete and filtered as proposed by Wang et al), and MSigDB pathways. Additionally the results of the TANGO analysis are attached. Pathways common both to van't Veer and one of Wang signatures are highlighted.
(XLS)

Table S3 Pathway overlap significance. The results of our suggested random background model, estimating overlap significance.
(XLS)

Table S4 The proliferation genes omitted. The genes were selected according to correlation in expression in the EMC-344 cohort to the genes MKI67, TOP2A or CDK1.
(XLS)

Table S5 Enrichment analysis after omitting proliferation genes. Hypergeometric enrichment of the signatures minus the genes that correlated with proliferation.
(XLS)

Table S6 Pathway overlap significance after omitting proliferation genes. Same as Table S2, but calculated after omitting proliferation genes.
(XLS)
